# Supplementary material for: Sec24 phosphorylation regulates autophagosome abundance during nutrient deprivation
Source: eLife. 2016 Nov 18;5:e21167. doi: 10.7554/eLife.21167 (PMC5148606; doi:10.7554/eLife.21167)
Supplement: Supplementary file 2. — DOI: http://dx.doi.org/10.7554/eLife.21167.027 [file elife-21167-supp2.docx]

Supplementary File 2. Key yeast strains used in this study.

| **Strain Number** | **Genotype** | **Source** |
| --- | --- | --- |
| SFNY 1948 | *MATa ade2-101 his3-Δ200 leu2-Δ1 lys2-801am trp1-Δ63 ura3-52 sec23Δ::His3MX6 (SEC23, URA3, CEN)* | Ferro-Novick Lab Collection |
| SFNY 2181 | *MATa GAL+ ura3-52 leu2-3,112 his3-Δ200 pSFNB1894 (GAL1-SEC23, URA3, CEN) pSFNB1895 (GAL1-SEC24-His, LEU2, CEN)* | Ferro-Novick Lab Collection |
| SFNY 2201 | *MATa sec24Δ::LEU2 ade2-1 can1-100 his3-11,15 trp1-1 leu2-3,112 ura3-1 pLM22 (SEC24, URA3, CEN)* | Ferro-Novick Lab Collection |
| SFNY 2202 | *MATα sec24Δ::LEU2 iss1Δ::KanMX ade2-1 can1-100 his3-11,15 trp1-1 leu2-3,112 ura3-1 pLM22 (SEC24, URA3, CEN)* | Ferro-Novick Lab Collection |
| SFNY 2287 | *MATα sec24Δ::LEU2 iss1Δ::KanMX ade2-1 can1-100 his3-11,15 trp1-1 leu2-3,112 ura3-1 pSFNB1915 (SEC24, HIS3, CEN)* | Ferro-Novick Lab Collection |
| SFNY 2366 | *MATα sec24Δ::LEU2 iss1Δ::KanMX can1-100 his3-11,15 trp1-1 leu2-3,112 ura3-1 pSFNB2054 (SEC24 T324A/T325A/T328A, HIS3, CEN)* | This study |
| SFNY 2367 | *MATa ade2-1 his3-11,15 leu2-3,112 trp1-1 ura3-1 PEP4::TRP1* | Ferro-Novick Lab Collection |
| SFNY 2368 | *MATa ade2-1 his3-11,15 leu2-3,112 trp1-1 ura3-1 PEP4::TRP1, pLM2 (GAL1-SEC24-His, LEU2, CEN), pTKY9 (GAL1-SEC23, URA3, CEN)* | Ferro-Novick Lab Collection |
| SFNY 2472 | *MATa ade2-1 his3-11,15 leu2-3,112 trp1-1 ura3-1 PEP4::TRP1 pSFNB2144 (GAL1-SEC24(S645A/S678A)-His, LEU2, CEN) pTKY9 (GAL1-SEC23, URA3, CEN)* | This study |
| SFNY 2473 | *MATa ade2-1 his3-11,15 leu2-3,112 trp1-1 ura3-1 PEP4::TRP1 pSFNB2143 (GAL1-SEC24(S645D/S678D)-His, LEU2, CEN) pTKY9 (GAL1-SEC23, URA3, CEN)* | This study |
| SFNY 2539 | *MATα sec24Δ::LEU2 iss1Δ::KanMX ade2-1 can1-100 his3-11,15 trp1-1 leu2-3,112 ura3-1 pho8::pho8Δ60 pSFNB1915 (SEC24, HIS3, CEN)* | This study |
| SFNY 2540 | *MATα sec24Δ::LEU2 iss1Δ::KanMX ade2-1 can1-100 his3-11,15 trp1-1 leu2-3,112 ura3-1 pho8::pho8Δ60 pSFNB1970 (SEC24 S730D/S735D, HIS3, CEN)* | This study |
| SFNY 2541 | *MATα sec24Δ::LEU2 iss1Δ::KanMX ade2-1 can1-100 his3-11,15 trp1-1 leu2-3,112 ura3-1 pho8::pho8Δ60 pSFNB1975 (SEC24 S730A/S735A, HIS3, CEN)* | This study |
| SFNY 2544 | *MATα sec24Δ::LEU2 iss1Δ::KanMX ade2-1 can1-100 his3-11,15 trp1-1 leu2-3,112 ura3-1 pho8::pho8Δ60 pSFNB2117 (SEC24 T324A/T325A, HIS3, CEN)* | This study |
| SFNY 2548 | *MATα sec24Δ::LEU2 iss1Δ::KanMX ade2-1 can1-100 his3-11,15 trp1-1 leu2-3,112 ura3-1 pho8::pho8Δ60 pSFNB2054 (SEC24 T324A/T325A/T328A, HIS3, CEN)* | This study |
| SFNY 2549 | *MATα sec24Δ::LEU2 iss1Δ::KanMX ade2-1 can1-100 his3-11,15 trp1-1 leu2-3,112 ura3-1 pho8::pho8Δ60 pSFNB2067 (SEC24 T328E, HIS3, CEN)* | This study |
| SFNY 2553 | *MATα sec24Δ::LEU2 iss1Δ::KanMX ade2-1 can1-100 his3-11,15 trp1-1 leu2-3,112 ura3-1 pho8::pho8Δ60 pSFNB2118 (SEC24 T325A/T328A, HIS3, CEN)* | This study |
| SFNY 2554 | *MATa ade2-101 his3-Δ200 leu2-Δ1 lys2-801am trp1-Δ63 ura3-52 sec23Δ::His3MX6 pho8::pho8Δ60 pCF364 (SEC23, TRP1, CEN)* | Ferro-Novick Lab Collection |
| SFNY 2557 | *MATa sec24Δ::LEU2 can1-100 his3-11,15 trp1-1 leu2-3,112 ura3-1 pho8::pho8Δ60 pSFNB1915 (SEC24, HIS3, CEN)* | This study |
| SFNY 2560 | *MATa sec24Δ::LEU2 can1-100 his3-11,15 trp1-1 leu2-3,112 ura3-1 pho8::pho8Δ60 pSFNB2102 (SEC24 T324E/T325E, HIS3, CEN)* | This study |
| SFNY 2574 | *MATα his3Δ1 leu2Δ0 ura3Δ0 met15Δ0 hrr25Δ::KanMX6 ATG2-GFP::His3MX6 pSFNB1715 (HRR25, LEU2, CEN) pSFNB2194 (APE1-RFP, URA3, CEN)* | This study |
| SFNY 2575 | *MATα his3Δ1 leu2Δ0 ura3Δ0 met15Δ0 hrr25Δ::KanMX6 ATG2-GFP::His3MX6 pSFNB1871 (hrr25-5, LEU2, CEN) pSFNB2194 (APE1-RFP, URA3, CEN)* | This study |
| SFNY 2576 | *MATα his3Δ1 leu2Δ0 ura3Δ0 met15Δ0 hrr25Δ::KanMX6 ATG13-GFP::His3MX6 pSFNB1715 (HRR25, LEU2, CEN) pSFNB2194 (APE1-RFP, URA3, CEN)* | This study |
| SFNY 2577 | *MATα his3Δ1 leu2Δ0 ura3Δ0 met15Δ0 hrr25Δ::KanMX6 ATG13-GFP::His3MX6 pSFNB1871 (hrr25-5, LEU2, CEN) pSFNB2194 (APE1-RFP, URA3, CEN)* | This study |
| SFNY 2578 | *MATα his3Δ1 leu2Δ0 ura3Δ0 met15Δ0 hrr25Δ::KanMX6 ATG14-GFP::His3MX6 pSFNB1715 (HRR25, LEU2, CEN) pSFNB2194 (APE1-RFP, URA3, CEN)* | This study |
| SFNY 2579 | *MATα his3Δ1 leu2Δ0 ura3Δ0 met15Δ0 hrr25Δ::KanMX6 ATG14-GFP::His3MX6 pSFNB1871 (hrr25-5, LEU2, CEN) pSFNB2194 (APE1-RFP, URA3, CEN)* | This study |
| SFNY 2624 | *MATa ade2-101 his3-Δ200 leu-2Δ1 lys2-801am trp1-Δ63 ura3-52 sec23Δ::His3MX6 pCF364 (SEC23, TRP1, CEN) pSFNB1637 (GFP-ATG8, URA3, CEN)* | Ferro-Novick Lab Collection |
| SFNY 2627 | *MATα ura3-52 leu2-3,112 his3-Δ200 SEC13-GFP::URA3 pSFNB2193 (APE1-RFP, LEU2, CEN)* | Ferro-Novick Lab Collection |
| SFNY 2633 | *MATα sec24Δ::LEU2 iss1Δ::KanMX ade2-1 can1-100 his3-11,15 trp1-1 leu2-3,112 ura3-1 pSFNB1915 (SEC24, HIS3, CEN) pSFNB1637 (GFP-ATG8, URA3, CEN)* | This study |
| SFNY 2634 | *MATα sec24Δ::LEU2 iss1Δ::KanMX ade2-1 can1-100 his3-11,15 trp1-1 leu2-3,112 ura3-1 pSFNB1975 (SEC24 S730A/S735A, HIS3, CEN) pSFNB1637 (GFP-ATG8, URA3, CEN)* | This study |
| SFNY 2635 | *MATα sec24Δ::LEU2 iss1Δ::KanMX ade2-1 can1-100 his3-11,15 trp1-1 leu2-3,112 ura3-1 pSFNB1970 (SEC24 S730D/S735D, HIS3, CEN) pSFNB1637 (GFP-ATG8, URA3, CEN)* | This study |
| SFNY 2636 | *MATa sec24Δ::LEU2 can1-100 his3-11,15 trp1-1 leu2-3,112 ura3-1 pSFNB1915 (SEC24, HIS3, CEN) pSFNB1637 (GFP-ATG8, URA3, CEN)* | This study |
| SFNY 2638 | *MATa sec24Δ::LEU2 can1-100 his3-11,15 trp1-1 leu2-3,112 ura3-1 pSFNB2054 (SEC24 T324A/T325A/T328A, HIS3, CEN) pSFNB1637 (GFP-ATG8, URA3, CEN)* | This study |
| SFNY 2649 | *MATa ura3-1 his3-11,15 trp1-1 leu2-3,112 ade2-1 can1-100 pBY924 (HRR25-HA, URA3, CEN)* | This study |
| SFNY 2653 | *MATα sec24Δ::LEU2 iss1Δ::KanMX ade2-1 can1-100 his3-11,15 trp1-1 leu2-3,112 ura3-1 pSFNB2054 (SEC24 T324A/T325A/T328A, HIS3, CEN) pSFNB1637 (GFP-ATG8, URA3, CEN)* | This study |
| SFNY 2654 | *MATα his3Δ1 leu2Δ0 ura3Δ0 met15Δ0 ATG9-13myc::URA3 hrr25Δ::KAN pSFNB1715 (HRR25, LEU2, CEN)* | This study |
| SFNY 2655 | *MATα his3Δ1 leu2Δ0 ura3Δ0 met15Δ0 ATG9-13myc::URA3 hrr25Δ::KAN pSFNB1871 (hrr25-5, LEU2, CEN)* | This study |
| SFNY 2686 | *MATa ade2-1 his3-11,15 leu2-3,112 trp1-1 ura3-1 PEP4::TRP1 pSFNB1973 (GAL1-SEC24(S730D/S735D)-His, LEU2, CEN) pTKY9 (GAL1-SEC23, URA3, CEN)* | This study |
| SFNY 2687 | *MATa ade2-1 his3-11,15 leu2-3,112 trp1-1 ura3-1 PEP4::TRP1 pSFNB1977 (GAL1-SEC24(S730A/S735A)-His, LEU2, CEN) pTKY9 (GAL1-SEC23, URA3, CEN)* | This study |
| SFNY 2738 | *MATa ade2-101 his3-Δ200 leu2-Δ1 lys2-801am trp1-Δ63 ura3-52 sec23Δ::His3MX6 pho8::pho8Δ60 pSFNB2048 (SEC23 T146E/S147D/S149D, TRP1, CEN)* | This study |
| SFNY 2740 | *MATa ade2-101 his3-Δ200 leu2-Δ1 lys2-801am trp1-Δ63 ura3-52 sec23Δ::His3MX6 pho8::pho8Δ60 pSFNB2273 (SEC23 T146A/S147A/S149A, TRP1, CEN)* | This study |
| SFNY 2769 | *MATα sec24Δ::LEU2 iss1Δ::KanMX ade2-1 can1-100 his3-11,15 trp1-1 leu2-3,112 ura3-1 ATG9-13myc::URA3 pSFNB1915 (SEC24, HIS3, CEN)* | This study |
| SFNY 2770 | *MATα sec24Δ::LEU2 iss1Δ::KanMX ade2-1 can1-100 his3-11,15 trp1-1 leu2-3,112 ura3-1 ATG9-13myc::URA3 pSFNB2054 (SEC24 T324A/T325A/T328A, HIS3, CEN)* | This study |
| SFNY 2771 | *MATα sec24Δ::LEU2 iss1Δ::KanMX ade2-1 can1-100 his3-11,15 trp1-1 leu2-3,112 ura3-1 ATG9-13myc::URA3 pSFNB2117 (SEC24 T324A/T325A, HIS3, CEN)* | This study |
| SFNY 2772 | *MATa sec24Δ::LEU2 ade2-1 can1-100 his3-11,15 trp1-1 leu2-3,112 ura3-1 ATG9-13myc::URA3 pSFNB1915 (SEC24, HIS3, CEN)* | This study |
| SFNY 2773 | *MATa sec24Δ::LEU2 ade2-1 can1-100 his3-11,15 trp1-1 leu2-3,112 ura3-1 ATG9-13myc::URA3 pSFNB2102 (SEC24 T324E/T325E, URA3, CEN)* | This study |
| SFNY 2778 | *MATα sec24Δ::LEU2 iss1Δ::KanMX ade2-1 can1-100 his3-11,15 trp1-1 leu2-3,112 ura3-1 pho8::pho8Δ60 pSFNB2208 (SEC24 T324A, HIS3, CEN)* | This study |
| SFNY 2779 | *MATα sec24Δ::LEU2 iss1Δ::KanMX ade2-1 can1-100 his3-11,15 trp1-1 leu2-3,112 ura3-1 pho8::pho8Δ60 pSNFB2209 (SEC24 T325A, HIS3, CEN)* | This study |
| SFNY 2780 | *MATα sec24Δ::LEU2 iss1Δ::KanMX ade2-1 can1-100 his3-11,15 trp1-1 leu2-3,112 ura3-1 pho8::pho8Δ60 pSFNB2210 (SEC24 T328A, HIS3, CEN)* | This study |
| SFNY 2781 | *MATα sec24Δ::LEU2 iss1Δ::KanMX ade2-1 can1-100 his3-11,15 trp1-1 leu2-3,112 ura3-1 pho8::pho8Δ60 pSFNB2211 (SEC24 T324A/T328A, HIS3, CEN)* | This study |
| SFNY 2782 | *MATα sec24Δ::LEU2 iss1Δ::KanMX ade2-1 can1-100 his3-11,15 trp1-1 leu2-3,112 ura3-1 pho8::pho8Δ60 pSFNB2087 (SEC24 T325E, HIS3, CEN)* | This study |
| SFNY 2785 | *MATa ura3–52 pSFNB1637 (GFP-ATG8, URA3, CEN)* | This study |
| SFNY 2786 | *MATa ura3-52 sec12-4 pSFNB1637 (GFP-ATG8, URA3, CEN)* | This study |
| SFNY 2787 | *MATα ura3-52 leu2-3,112 his3-Δ200 pSFNB1637 (GFP-ATG8, URA3, CEN)* | This study |
| SFNY 2788 | *MATα ura3-52 leu2-3,112 his3-Δ200 atg11Δ::His3MX6 pSFNB1637 (GFP-ATG8, URA3, CEN)* | This study |
| SFNY 2783 | *MATα sec24Δ::LEU2 iss1Δ::KanMX ade2-1 can1-100 his3-11,15 trp1-1 leu2-3,112 ura3-1 pho8::pho8Δ60 pSFNB2101 (SEC24 T324E/T328E, HIS3, CEN)* | This study |
| SFNY 2789 | *MATα sec24Δ::LEU2 iss1Δ::KanMX ade2-1 can1-100 his3-11,15 trp1-1 leu2-3,112 ura3-1 ATG2-3xGFP::CaURA3 pSFNB1915 (SEC24, HIS3, CEN) pSFNB2261 (APE1-RFP, TRP1, CEN)* | This study |
| SFNY 2790 | *MATα sec24Δ::LEU2 iss1Δ::KanMX ade2-1 can1-100 his3-11,15 trp1-1 leu2-3,112 ura3-1 ATG2-3xGFP::CaURA3 pSFNB2054 (SEC24 T324A/T325A/T328A, HIS3, CEN) pSFNB2261 (APE1-RFP, TRP1, CEN)* | This study |
| SFNY 2791 | *MATα sec24Δ::LEU2 iss1Δ::KanMX ade2-1 can1-100 his3-11,15 trp1-1 leu2-3,112 ura3-1 ATG5-3xGFP::CaURA3 pSFNB1915 (SEC24, HIS3, CEN) pSFNB2261 (APE1-RFP, TRP1, CEN)* | This study |
| SFNY 2792 | *MATα sec24Δ::LEU2 iss1Δ::KanMX ade2-1 can1-100 his3-11,15 trp1-1 leu2-3,112 ura3-1 ATG5-3xGFP::CaURA3 pSFNB2054 (SEC24 T324A/T325A/T328A, HIS3, CEN) pSFNB2261 (APE1-RFP, TRP1, CEN)* | This study |
| SFNY 2793 | *MATα sec24Δ::LEU2 iss1Δ::KanMX ade2-1 can1-100 his3-11,15 trp1-1 leu2-3,112 ura3-1 ATG9-3xGFP::CaURA3 pSFNB1915 (SEC24, HIS3, CEN) pSFNB2261 (APE1-RFP, TRP1, CEN)* | This study |
| SFNY 2794 | *MATα sec24Δ::LEU2 iss1Δ::KanMX ade2-1 can1-100 his3-11,15 trp1-1 leu2-3,112 ura3-1 ATG9-3xGFP::CaURA3 pSFNB2054 (SEC24 T324A/T325A/T328A, HIS3, CEN) pSFNB2261 (APE1-RFP, TRP1, CEN)* | This study |
| SFNY 2795 | *MATα sec24Δ::LEU2 iss1Δ::KanMX ade2-1 can1-100 his3-11,15 trp1-1 leu2-3,112 ura3-1 ATG13-3xGFP::CaURA3 pSFNB1915 (SEC24, HIS3, CEN) pSFNB2261 (APE1-RFP, TRP1, CEN)* | This study |
| SFNY 2796 | *MATα sec24Δ::LEU2 iss1Δ::KanMX ade2-1 can1-100 his3-11,15 trp1-1 leu2-3,112 ura3-1 ATG13-3xGFP::CaURA3 pSFNB2054 (SEC24 T324A/T325A/T328A, HIS3, CEN) pSFNB2261 (APE1-RFP, TRP1, CEN)* | This study |
| SFNY 2797 | *MATα sec24Δ::LEU2 iss1Δ::KanMX ade2-1 can1-100 his3-11,15 trp1-1 leu2-3,112 ura3-1 ATG14-3xGFP::CaURA3 pSFNB1915 (SEC24, HIS3, CEN) pSFNB2261 (APE1-RFP, TRP1, CEN)* | This study |
| SFNY 2798 | *MATα sec24Δ::LEU2 iss1Δ::KanMX ade2-1 can1-100 his3-11,15 trp1-1 leu2-3,112 ura3-1 ATG14-3xGFP::CaURA3 pSFNB2054 (SEC24 T324A/T325A/T328A, HIS3, CEN) pSFNB2261 (APE1-RFP, TRP1, CEN)* | This study |
| SFNY 2799 | *MATα his3Δ1 leu2Δ0 ura3Δ0 met15Δ0 hrr25Δ::KanMX6 ATG5-GFP::His3MX6 pSFNB2194 (APE1-RFP, URA3, CEN) pSFNB1715 (HRR25, LEU2, CEN)* | This study |
| SFNY 2800 | *MATα his3Δ1 leu2Δ0 ura3Δ0 met15Δ0 hrr25Δ::KanMX6 ATG5-GFP::His3MX6 pSFNB2194 (APE1-RFP, URA3, CEN) pSFNB1871 (hrr25-5, LEU2, CEN)* | This study |
| SFNY 2801 | *MATα his3Δ1 leu2Δ0 ura3Δ0 met15Δ0 hrr25Δ::KanMX6 ATG9-GFP::His3MX6 pSFNB2194 (APE1-RFP, URA3, CEN) pSFNB1715 (HRR25, LEU2, CEN)* | This study |
| SFNY 2802 | *MATα his3Δ1 leu2Δ0 ura3Δ0 met15Δ0 hrr25Δ::KanMX6 ATG9-GFP::His3MX6 pSFNB2194 (APE1-RFP, URA3, CEN) pSFNB1871 (hrr25-5, LEU2, CEN)* | This study |
| SFNY 2809 | *MATa ura3-52 ATG2-GFP::KanMX6 pSFNB2194 (APE1-RFP, URA3, CEN)* | This study |
| SFNY 2810 | *MATa ura3-52 sec12-4 ATG2-GFP::KanMX6 pSFNB2194 (APE1-RFP, URA3, CEN)* | This study |
| SFNY 2811 | *MATa ura3-52 ATG14-GFP::KanMX6 pSFNB2194 (APE1-RFP, URA3, CEN)* | This study |
| SFNY 2812 | *MATa ura3-52 sec12-4 ATG14-GFP::KanMX6 pSFNB2194 (APE1-RFP, URA3, CEN)* | This study |
| SFNY 2821 | *MATa ade2-101 his3-Δ200 leu2-Δ1 lys2-801am trp1-Δ63 ura3-52 sec23Δ::His3MX6 pSFNB2048 (SEC23 T146E/S147D/S149D, TRP1, CEN) pSNFB1637 (GFP-ATG8, URA3, CEN)* | This study |
| SFNY 2822 | *MATa ade2-101 his3-Δ200 leu2-Δ1 lys2-801am trp1-Δ63 ura3-52 sec23Δ::His3MX6 pSFNB2273 (SEC23 T146A/S147A/S149A, TRP1, CEN) pSFNB1637 (GFP-ATG8, URA3, CEN)* | This study |
| SFNY 2989 | *MATα sec24Δ::LEU2 iss1Δ::KanMX pep4Δ::TRP1 ade2-1 can1-100 his3-11,15 trp1-1 leu2-3,112 ura3-1 pSFNB1915 (SEC24, HIS3, CEN)* | This study |
| SFNY 2990 | *MATα sec24Δ::LEU2 iss1Δ::KanMX pep4Δ::TRP1 ade2-1 can1-100 his3-11,15 trp1-1 leu2-3,112 ura3-1 pSFNB2054 (SEC24 T324A/T325A/T328A, HIS3, CEN)* | This study |
| SFNY 2999 | *MATa GAL+ ura3-52 leu2-3,112 his3-Δ200 ATG9-13myc::LEU2 ypt7Δ::His3MX6* | This study |
| SFNY 3037 | *MATα his3Δ1 leu2Δ0 ura3Δ0 met15Δ0 hrr25Δ::KanMX6 pep4Δ::His3MX6 pSFNB1715 (HRR25, LEU2, CEN)* | This study |
| SFNY 3038 | *MATα his3Δ1 leu2Δ0 ura3Δ0 met15Δ0 hrr25Δ::KanMX6 pep4Δ::His3MX6 pSFNB1871 (hrr25-5, LEU2, CEN)* | This study |
| SFNY 3058 | *MATa ade2-1 his3-11,15 leu2-3,112 trp1-1 ura3-1 PEP4::TRP1, pSFNB2301 (GAL1-SEC24(T324E/T328E)-His, LEU2, CEN), pTKY9 (GAL1-SEC23, URA3, CEN)* | This study |
| SFNY 3060 | *MATa ade2-1 his3-11,15 leu2-3,112 trp1-1 ura3-1 PEP4::TRP1, pSFNB2303 (GAL1-SEC24(T325E)-His, LEU2, CEN), pTKY9 (GAL1-SEC23, URA3, CEN)* | This study |
| SFNY 3063 | *MATα his3Δ1 leu2Δ0 ura3Δ0 met15Δ0 hrr25Δ::KanMX6 pSFNB1871 (hrr25-5, LEU2, CEN) pSFNB2118 (SEC24 T325A/T328A, HIS3, CEN) pSFNB1637 (GFP-ATG8, URA3, CEN)* | This study |
| SFNY 3065 | *MATα his3Δ1 leu2Δ0 ura3Δ0 met15Δ0 hrr25Δ::KanMX6 ATG9-13myc::URA3 pSFNB1871 (hrr25-5, LEU2, CEN) pSFNB2118 (SEC24 T325A/T328A, HIS3, CEN)* | This study |
| SFNY 3078 | *MATα his3Δ1 leu2Δ0 ura3Δ0 met15Δ0 hrr25Δ::KanMX6 pSFNB1871 (hrr25-5, LEU2, CEN) pSFNB1915 (SEC24, HIS3, CEN) pSFNB1637 (GFP-ATG8, URA3, CEN)* | This study |
| SFNY 3080 | *MATα his3Δ1 leu2Δ0 ura3Δ0 met15Δ0 hrr25Δ::KanMX6 pSFNB1871 (hrr25-5, LEU2, CEN) pSFNB2103 (SEC24 T325E/T328E, HIS3, CEN) pSFNB1637 (GFP-ATG8, URA3, CEN)* | This study |
| SFNY 3081 | *MATα his3Δ1 leu2Δ0 ura3Δ0 met15Δ0 hrr25Δ::KanMX6 pSFNB1715 (HRR25, LEU2, CEN) pSFNB1915 (SEC24, HIS3, CEN) pSFNB1637 (GFP-ATG8, URA3, CEN)* | This study |
| SFNY 3082 | *MATα his3Δ1 leu2Δ0 ura3Δ0 met15Δ0 hrr25Δ::KanMX6 ATG9-13myc::URA3 pSFNB1871 (hrr25-5, LEU2, CEN) pSFNB1915 (SEC24, HIS3, CEN)* | This study |
| SFNY 3084 | *MATα his3Δ1 leu2Δ0 ura3Δ0 met15Δ0 hrr25Δ::KanMX6 ATG9-13myc::URA3 pSFNB1871 (hrr25-5, LEU2, CEN) pSFNB2103 (SEC24 T325E/T328E, HIS3, CEN)* | This study |
| SFNY 3085 | *MATα his3Δ1 leu2Δ0 ura3Δ0 met15Δ0 hrr25Δ::KanMX6 ATG9-13myc::URA3 pSFNB1715 (HRR25, LEU2, CEN) pSFNB1915 (SEC24, HIS3, CEN)* | This study |
| SFNY 3095 | *MATα ura3-52 leu2-3,112 his3-Δ200 atg9Δ::His3MX6 SEC13-GFP::URA3 pSFNB2193 (APE1-RFP, LEU2, CEN)* | This study |
